# Succinate Is a Natural Suppressor of Antiviral Immune Response by Targeting MAVS

**DOI:** 10.3389/fimmu.2022.816378

**Published:** 2022-03-02

**Authors:** Yue Xiao, Xinyi Chen, Zhun Wang, Jiazheng Quan, Xibao Zhao, Haimei Tang, Han Wu, Qianqian Di, Zherui Wu, Weilin Chen

**Affiliations:** ^1^Department of Immunology, School of Medicine, Shenzhen University, Shenzhen, China; ^2^Technological Center, Changchun Customs, Changchun, China

**Keywords:** succinate, metabolism, VSV, antiviral immune response, MAVS

## Abstract

Succinate is at the crossroads of multiple metabolic pathways and plays a role in several immune responses acting as an inflammation signal. However, whether succinate regulates antiviral immune response remains unclear. Here, we found that the production of succinate was reduced in RAW264.7 cells during vesicular stomatitis virus (VSV) infection. Using diethyl succinate to pretreat the mouse peritoneal macrophages and RAW264.7 cells before VSV infection, the production of interferon-β (IFN-β), chemokine (C–X–C motif) ligand 10 (CXCL-10), and IFN-stimulated genes 15 (ISG15) was significantly decreased, following which the VSV replication in diethyl succinate-pretreated cells was obviously increased. Moreover, succinate decreased the expression of IFN-β in serum, lung, and spleen derived from the VSV-infected mice. The overall survival rate in the VSV-infected mice with diethyl succinate pretreatment was also remarkably downregulated. Furthermore, we identified that succinate inhibited the activation of MAVS-TBK1-IRF3 signaling by suppressing the formation of MAVS aggregates. Our findings provide previously unrecognized roles of succinate in antiviral immune response and establish a novel link between metabolism and innate immune response.

## Introduction

Cellular metabolism is now considered a prominent route in our quest to understand immune responses (1). In recent years, the Krebs cycle metabolite succinate has been in succession reported that concerned with immune responses. Succinate could induce migratory responses of dendritic cells and act in synergy with Toll-like receptor ligands for proinflammatory cytokine production through binding to succinate receptor SUNCR1, also known as G protein–coupled receptor (GPR91) (2). SUNCR1 is expressed on various types of cells including immune cells (3). *Via* the succinate-SUNCR1 signaling axis, succinate triggered pro-inflammatory response in macrophages during antigen-induced arthritis and induced a type 2 immune response in the small intestine (4–6). In the lipopolysaccharide (LPS)-stimulated macrophages, succinate acted as a metabolite in innate immune signaling to enhance interleukin-1β production through hypoxia-inducible factor-1α (7). However, whether succinate has a role in antiviral immune response remains unclear.

Type I interferons (IFNs) produced by nearly all cell types play a pivotal role in host defense against viral infection. Type I IFNs, including IFN-α, IFN-β, and other IFN family members, are induced by the activation of cell-surface or intracellular pattern recognition receptors (PRRs) (8). During RNA viral infection, cytoplasmic RNA species are recognized by retinoic-acid inducible gene I (RIG-I)-like receptors (RLRs, a member of PRRs) composed of RIG-I and melanoma differentiation-associated gene 5 (MDA5). RIG-I or MDA5 detects and binds with viral RNA *via* their C-terminal repressor (9). Upon sensing RNA, the activation of RIG-I and MDA5 undergoes a conformational change to recruit mitochondrial antiviral signaling (MAVS) protein. The C-terminus transmembrane domain of MAVS is necessary for its localization in the mitochondrial outer membrane. Once activated, MAVS develops a functional prion-like structure at the mitochondria and activates the downstream protein kinase TBK1, resulting in phosphorylation of the transcription factor interferon regulatory factor 3 (IRF3) and its nuclear translocation to drive the production of type I IFN (8–11). Intracellular RIG-I-MAVS signaling and type I IFN production are orchestrated by numerous mechanisms (12–14). Recently, energy metabolism in the regulation of antiviral innate immunity is becoming the new research field to understand the host antiviral immune response. Zhang et al. demonstrated that glucose-generated lactate acts as a regulator of MAVS to inhibit RLR signaling, allowing a cross-regulation between antiviral signaling and energy metabolism (15). The study, which showed that hexosamine biosynthesis pathway (HBP)-associated *O*-linked β-N-acetylglucosamine (*O*-GlcNAc) mediated the O-GlcNacylation and stabilization of sterile alpha motif and histidine/aspartic acid domain-containing protein 1 (SAMHD1) to positively regulate host antiviral response against HBV, revealed a link between HBP and innate antiviral immunity further (16). Therefore, it is necessary to explore more biochemical regulations between energy metabolism and antiviral response for creating exciting novel opportunities in therapeutic applications for virus infection in future.

In this study, using the mouse peritoneal macrophages and RAW264.7 cell culture systems, as well as a VSV-infection mouse model, we found that succinate negatively regulates the innate immunity in response to VSV infection *in vitro* and *in vivo*. Our results revealed that succinate could inhibit aggregation of MAVS and its activation. These findings suggest a vital role of succinate in limiting RLR signaling through targeting MAVS.

## Materials and Methods

### Mice and Cell Culture

C57BL/6 mice (female, 6- to 8-week-old) were purchased from the Guangdong Medical Laboratory Animal Center (Guangdong, China) and housed in SPF breeding units. The animal experimental manipulation was performed according to the National Institute of Health Guide for the Care and Use of Laboratory Animals with the approval of Shenzhen University (Approval Number: AEWC-20200316).

Mouse peritoneal macrophages (MPMs) were harvested from mice which had received a 2-ml intraperitoneal injection of 3% Brewer thioglycollate broth (Sigma, Poole, UK) 3 days prior to collection. The MPMs were cultured in RPMI 1640 with 10% FCS. Mouse macrophage cell line RAW264.7 cells and HEK293T cells were purchased from ATCC and cultured in DMEM medium containing 10% FBS.

### Plasmids, Antibodies, and Reagents

Myc-tagged RIG-I (N), Myc-tagged MAVS, Myc-tagged TBK1, and Myc-tagged IRF3-5D plasmids were constructed by cloning into the pcDNA3.1 vector. Antibodies against RIG-I (#3743), MAVS (#83000), TBK1 (#3504), IRF3 (#4302), Phospho-TBK1 (5483), and Phospho-IRF3 (#4947) were purchased from Cell Signaling Technology (Danvers, MA, USA). Succinic acid (#S9512) was purchased from Sigma-Aldrich (St. Louis, USA).

### Virus Infection and Reagent Treatment

Cells were infected with VSV (multiplicity of infection (MOI) = 1) or VSV-eGFP (MOI = 0.01) for the indicated hours. For *in vivo* infection, the regular dose of VSV for mice was 1× 10^8^ PFU/g by intraperitoneal injection and the half-lethal dose was 1.3 × 10^7^ PFU/g by intravenous injection. The doses of dimethyl succinate (Ds) in cells and mice were 5 and 62.5 mM in 100 μl PBS per mouse separately.

### Cell Transfection and Dual-Luciferase Reporter Assay

Plasmids were transfected into HEK293T cells using jetPRIME transfection reagents according to the manufacturer’s protocol. Dual-luciferase reporter assay was performed in a 96-well plate with 90-ng luciferase reporter plasmids (IFN-β or ISRE luciferase reporter), 10 ng indicated Renilla luciferase construct phRL-TK (Promega, for normalizing the transfection efficiency), and 50 ng indicated expressing plasmids. The cells were harvested at 24 h post transfection. Luciferase assays were performed using a dual-specific luciferase assay kit (#E1960, Promega, Madison, WI, USA) in accordance with instructions. All samples were set as triplicate, and the folds were calculated relative to the baseline control in each experiment.

### Real-Time Quantitative PCR

The total RNA of cells was isolated by TRIzol reagent (Takara, Japan). Reverse transcription was used to generate cDNAs from RNA by Reverse Transcriptase M-MLV (RNase H-) (Takara, Shiga, Japan). Real-time quantitative PCR was performed with Hieff™ qPCR SYBR Green Master Mix (Yeasen, Shanghai, China) and detected with the Analytik Jena qTOWER^3^ PCR system (Jena, Germany). Data were determined by normalization of expression of β-actin in each sample and analyzed using the 2-ΔΔCT method. Gene-specific primer sequences were as the following: mouse β-actin, 5′-AGTGTGACGTTGACATCCGT-3′ and 5′-GCAGCTCAGTAACAGTCCGC-3′; mouse IFN-β, 5′-ATGAGTGGTGGTTGCAGGC-3′ and 5′-TGACCTTTCAAATGCAGTAGATTCA-3′; mouse GLUT1, 5′-TCATTGTCGGCCTCCTCATT-3′ and 5′-TAGGGTGGCAGAACTTGAGG-3′; mouse GLUT4, 5′-GCCATCGTCATTGGCATTCT-3′ and 5′-CTCCAGGTTCCGGATGATGT-3′; mouse IDH1, 5′-ACTCAGTCGCCCAAGGTTAT-3′ and 5′-GTAGTGACGTGTGACAGTGC-3′; mouse OGDH, 5′-CCACCCTGAGGCAAGAACTA-3′ and 5′-CCTTCCCAGTGCAGAAGAGA-3′.

### Western Blotting Assay

Cell lysates were prepared using RIPA buffer containing a protease inhibitor cocktail (Sigma-Aldrich, St. Louis, USA) and boiled with 1% SDS sample buffer. 20 to 40 μg of proteins was separated using 10% SDA-PAGE gels and then electro-transferred to nitrocellulose filter membranes (Millipore, Bedford, MA. USA). The membranes were blocked in 5% non-fat milk and immunoblotted with primary specific antibodies in concentrations as recommended by the manufacturer for overnight at 4°C. Secondary antibodies were conjugated with HRP. Blots were scanned using the Tanon 6100B Imaging System.

### Enzyme-Linked Immunosorbent Assay

RAW264.7 and peritoneal macrophage culture supernatants and mouse serum were collected and stored at -80°C. IFN-β was measured using a Mouse IFN-β ELISA Kit (BioLegend, San Diego, CA, USA) according to the manufacturer’s protocol.

### Flow Cytometric Assay

Cells were infected with VSV-eGFP or PBS for 16 h and underwent pancreatin digestion following twice cold PBS washing. Total cells were analyzed using Beckman CytoFLEX S. Results were analyzed by FlowJo version 10.4 software.

### Hematoxykin and Eosin Staining

Lungs from control or VSV-infected mice were fixed in 4% (weight/vol.) paraformaldehyde, embedded into paraffin, sectioned, stained with hematoxylin and eosin solution, and observed under the microscope.

### Semi-Denaturing Detergent Agarose Gel Electrophoresis

Semi-denaturing detergent agarose gel electrophoresis (SDD-AGE) was performed according to a published protocol with minor modifications (11) Briefly, equal amounts of the whole-cell lysate were resuspended in one-third volume of 4 × sample buffer (2 × TAE, 40% glycerol, 8% SDS, and 0.02% bromophenol blue). Samples were incubated for 5 min at room temperature and loaded onto a vertical 1.5% agarose gel containing a final concentration of 0.1% SDS. After electrophoresis in the running buffer (1 × TBE and 0.1% SDS) for 40 min with a constant voltage of 100 V at 4°C, proteins were transferred to PVDF membranes using a liquid transfer system in preparation for Western blotting analysis.

### Cellular Succinate Measurement

Intracellular succinate levels were measured using a Succinate Assay Kit (#ab204718, Abcam, Cambridge, USA) according to the manufacturer’s protocol. Briefly, 2 × 10^6^ cells/each assay were harvested and washed with cold PBS. Cells were resuspended in succinate assay buffer and homogenized quickly. Any insoluble material was removed by centrifugation. The collected supernatant was further deproteinized using a 10-kDa spin column (#ab93349, Abcam, Cambridge, USA) and subjected to succinate measurement.

### Statistical Analysis

All experiments assays were conducted at least three times with samples in triplicates. Statistical analysis comparisons between groups were determined by unpaired two-tailed Student’s *t*-test. p values < 0.05 were considered statistically significant. Animal survival curves were estimated for each group, the Kaplan–Meier method was adopted to generate graphs, and the survival curves were analyzed by log-rank analysis. GraphPad Prism 7.0 software (GraphPad Software, San Diego, CA) was used for all statistical analyses.

## Results

### VSV Infection Promotes Glucose Metabolism in Macrophages

**Figure 1:**
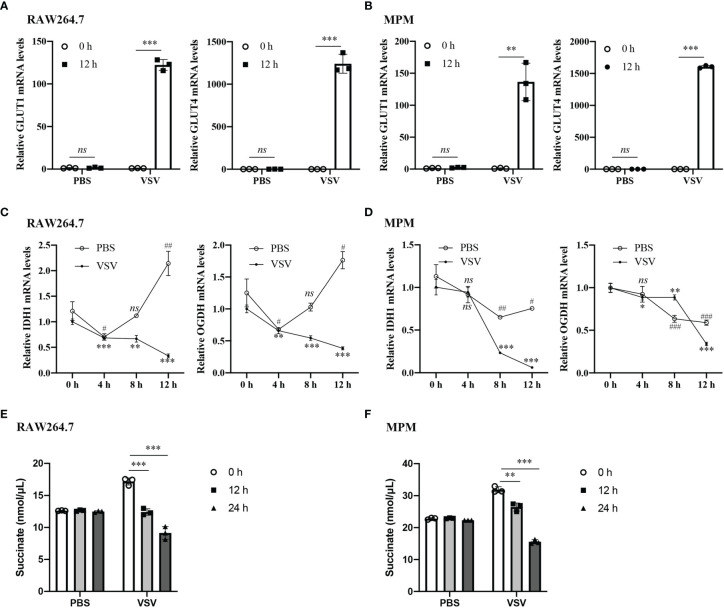
VSV infection promotes glucose metabolism in macrophages. **(A, B)** The mRNA levels of GLUT1 and GLUT4 were determined by RT-qPCR in RAW264.7 cells **(A)** and MPM **(B)** infected with vesicular stomatitis virus (VSV) (multiplicity of infection (MOI) = 1) or PBS for 12 h. **(C, D)** The mRNA levels of IDH1 **(C)** and OGDH **(D)** were determined by RT-qPCR in RAW264.7 cells **(C)** and MPM **(D)** infected with VSV (MOI = 1) or PBS for 4, 8, or 12 h. **(E, F)** Succinate abundance in RAW264.7 cells **(E)** and MPM **(F)** infected with VSV (MOI = 1) or PBS for 12 and 24 h. All data are represented as means ± SD. Significance calculated using the unpaired t-test (*vs. 0 h post VSV-infection; ^#^vs. 0 h post PBS-treatment), ^#^p < 0.05, ^##^p < 0.01, ^###^p < 0.001, **p < 0.01, ***p < 0.001, *ns: no significance*. Representative data are from three independent experiments.

To test glucose metabolic changes in response to viral challenge, we challenged RAW264.7 cells and mouse peritoneal macrophages (MPM) with vesicular stomatitis virus (VSV) or PBS as normal control and detected the expression levels of key markers in the glucose metabolic. RT-qPCR analysis showed that the mRNA levels of glucose transporter (GLUT) 1 and GLUT4 were significantly increased in the RAW264.7 cells and MPM infected with VSV for 12 h, while there were no changes in the PBS-treated group (**Figures 1A, B**). The mRNA levels of tricarboxylic acid (TCA) cycle key enzymes isocitrate dehydrogenase 1 (IDH1) and α-ketoglutarate dehydrogenase (OGDH) in RAW264.7 cells and MPM were gradually decreased with the prolongation of VSV infection time (**Figures 1C, D**). Besides, the Succinate Assay Kit (Colorimetric) was used to detect the TCA cycle intermediate succinate levels in the RAW264.7 cells; the results showed that succinate levels were also decreased in response to VSV infection (**Figures 1E, F**). These findings demonstrated that VSV infection could shift energy metabolism from oxidative phosphorylation (OXPHOS) to glycolysis in mouse macrophages.

### Succinate Negatively Regulates Virus-Induced IFN-β Production

**Figure 2:**
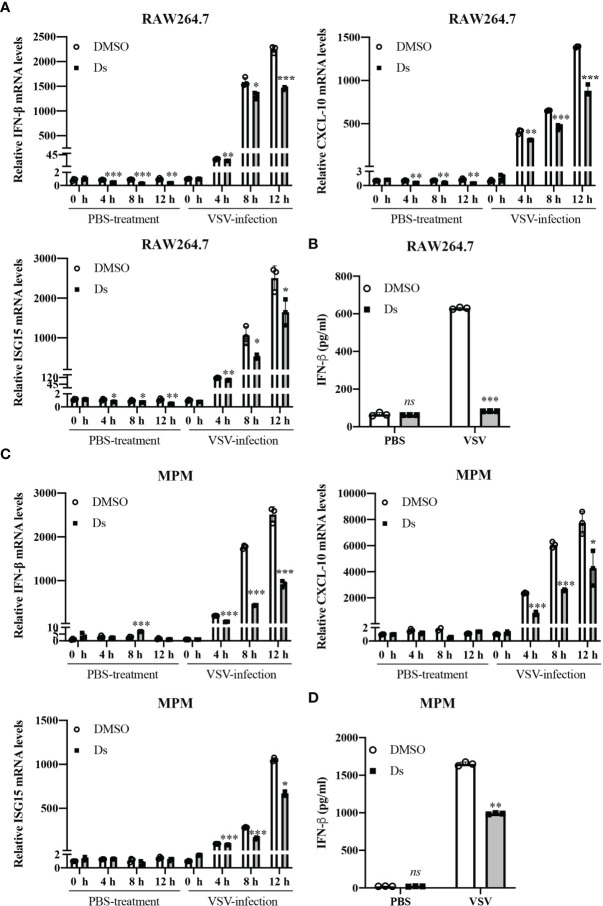
Succinate negatively regulates virus-induced IFN-β production. **(A, B)** RAW264.7 cells were pretreated with or without 5 mM diethyl succinate (Ds) for 3 h and then infected with VSV (MOI = 1) or PBS for 0, 4, 8, or 12 h. **(A)** The mRNA levels of IFN-β, CXCL-10, and ISG15 were measured by RT-qPCR in RAW264.7 cells. **(B)** The protein levels of IFN-β were measured by ELISA in RAW264.7 cells. **(C, D)** Mouse peritoneal macrophages (MPM) were pretreated with or without 5 mM diethyl succinate (Ds) for 3 h and then infected with VSV (MOI = 1) for 0, 4, 8, or 12 h. **(C)** The mRNA levels of IFN-β, CXCL-10, and ISG15 were measured by RT-qPCR in MPM cells. **(D)** The protein levels of IFN-β were measured by ELISA in MPM cells. All data are represented as means ± SD. Significance calculated using the unpaired *t*-test. *p < 0.05, **p < 0.01, ***p < 0.001. Representative data are from three independent experiments.

To explore the role of succinate in antiviral response, we used VSV to examine the effect of succinate on the production of IFN-β. RAW264.7 cells or MPM were pretreated with or without 5 mM cell-permeable diethyl succinate (Ds) for 3 h and then infected with VSV at multiplicity of infection (MOI) = 1 for 0, 4, 8, or 24 h. The cells treated with PBS were used as control group. The mRNA levels of IFN-β were lower in Ds-pretreated RAW264.7 cells than those in DMSO-pretreated cells (**Figure 2A**). Enzyme-linked immunosorbent assay (ELISA) showed that the protein level of IFN-β decreased by about 75% in the media of VSV-infected RAW264.7 cells cultured for 24 h in the presence of Ds (**Figure 2B**). Furthermore, in the Ds-pretreated MPM, the mRNA levels of IFN-β were significantly lower than those in the DMSO-pretreated MPM (**Figure 2C**). ELISA showed that IFN-β production decreased by about 43% in the media of VSV-infected MPM cultured for 24 h in the presence of Ds (**Figure 2D**). These results indicate that succinate inhibits the production of IFN-β in macrophages infected with VSV.

### Succinate Inhibits Cellular Antiviral Response in Macrophages

**Figure 3:**
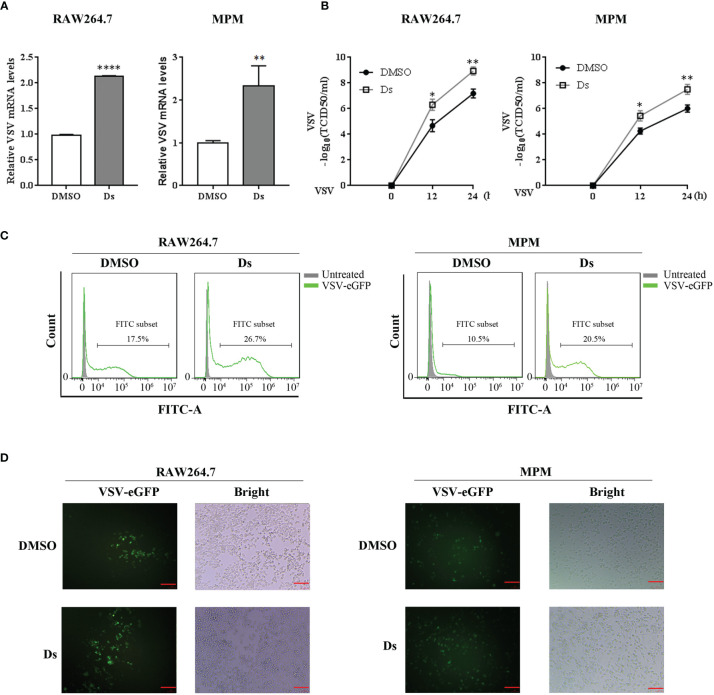
Succinate inhibits VSV replication in macrophages. RAW264.7 cells and MPM were pretreated with 5 mM Ds or DMSO for 3 h followed infection by VSV. **(A)** The mRNA levels of VSV were determined by RT-qPCR in RAW264.7 cells and MPM infected with VSV (MOI = 1) for 12 h. Data are represented as means ± SD. Significance calculated using the unpaired *t*-test. **p < 0.01, ****p < 0.0001. **(B)** VSV titers were determined by TCID50 assay in the supernatants of RAW264.7 cells and MPM with VSV (MOI = 1) challenged for 12 and 24 h. Data are represented as means ± SD. Significance calculated using the unpaired *t*-test. *p < 0.05, **p < 0.01. **(C)** The percentage of positive VSV-infected cells were determined by flow cytometry in RAW264.7 cells and MPM challenged with VSV-eGFP (MOI = 0.01) for 16 h. **(D)** RAW264.7 cells and MPM infected with VSV-eGFP (MOI = 0.01) that underwent fluorescence microscopy for 16 h, red scale bar = 100 μm. Representative data or images are from three independent experiments.

To investigate whether succinate affected cell antiviral ability, we treated RAW264.7 cells and MPM with Ds/DMSO before being infected with VSV. VSV replication and titer in cells were measured. As shown in **Figure 3A**, the mRNA levels of VSV in the Ds-pretreated cells with VSV infection for 12 h were higher than those in control cells. TCID50 assay showed that higher VSV titers in Ds-treated cells were detected in the supernatants of RAW264.7 cells and MPM with VSV infection for 12 and 24 h (**Figure 3B**). Furthermore, flow cytometry was used to confirm the role of succinate in VSV replication. The results revealed that 26.7% cells were infected (GFP+) in cells pretreated with Ds, compared to 17.5% of GFP-positive cells in DMSO-pretreated cells at 16 h post VSV-eGFP infection in RAW264.7 cells (**Figure 3C** left). There were 20.5% of GFP-positive cells in Ds-pretreated cells, compared to 10.5% of GFP-positive cells in DMSO-pretreated cells at 16 h post VSV-eGFP infection in MPM (**Figure 3C** right). Correspondingly, a fluorescence microscope was used to observe the positive cells infected by VSV-eGFP. The results showed that the RAW264.7 cells and MPM pretreated with Ds compared with control cells had more GFP-positive cells (**Figure 3D**). These data suggest that succinate inhibits cellular antiviral response.

### Succinate Suppressed the Antiviral Response of Mice *In Vivo*

**Figure 4:**
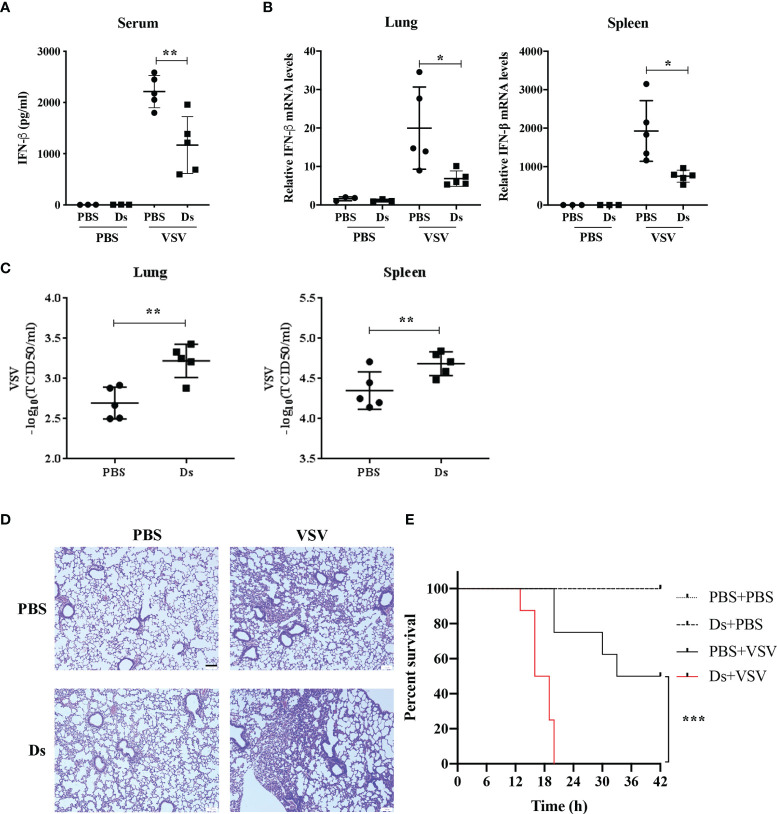
Succinate suppresses the antiviral response of mice *in vivo*. **(A–D)** Mice (6 weeks old) were infected with or without VSV (1 × 10^8^ PFU/g) *via* intraperitoneal injection for 12 h after Ds or PBS treatment for 3 h. VSV-non-infected group, n = 3/group; VSV-infected groups, n = 5/group. **(A)** The concentrations of IFN-β in serum from Ds or PBS-pretreated mice were detected by ELISA. **(B)** The IFN-β mRNA expression of lungs and spleens were determined by RT-qPCR. **(C)** The VSV loads in lungs and spleens were detected by TCID50. **(D)** Histological analysis of the lung tissue from mice in **(A)**, scale bar = 100 μm. Images are representative of three independent experiments. **(E)** Mice (6 weeks old, n = 8) pretreated with Ds or PBS for 3 h were injected with a half-lethal dose of VSV (1.3 × 10^7^ PFU/g) or PBS by tail intravenous injection, and survival was recorded for 42 h. Log-rank test, ***p < 0.001. **(A–C)** Data are shown as mean ± SD. Significance calculated using the unpaired *t*-test. *p < 0.05, **p < 0.01. Representative data are from three independent experiments.

To prove that succinate affected host antiviral innate response, we challenged mice with VSV (1 × 10^8^ PFU/g) (17) or PBS by intraperitoneal (i.p.) injection after Ds or PBS injection (i.p.) for 3 h. At 12 h post VSV infection, the serum, lungs, and spleens of mice were collected to test the IFN-β production and VSV loads. The results showed that the production of IFN-β in the serum from PBS-injected mice was nearly two-fold higher than in the serum from Ds-injected mice in the VSV-infected group (**Figure 4A**). The IFN-β mRNA levels of lungs and spleens from the Ds-injected mice were markedly reduced compared to PBS-injected mice in the VSV-infected group (**Figure 4B**). VSV titers in the lungs and spleens from the Ds-injected mice were lower than that in the PBS-injected mice (**Figure 4C**). H&E staining showed that the infiltration of inflammatory cells in the lung sections of PBS-injected mice was fewer than that in the Ds-injected mice after infection with VSV (**Figure 4D**). Furthermore, we studied the effects of succinate on the survival of VSV-infected mice. The Ds or PBS-pretreated mice (n = 8/group) were challenged with a half-lethal dose of VSV (1.3 × 10^7^ PFU/g) by intravenous (i.v.) injection, and the times to death of the mice were recorded. As shown in **Figure 4E**, at 20 h post VSV infection, all the mice were dead in the Ds-treated group; meanwhile, there were six mice still alive in the PBS-treated group. The survival assay showed that mice treated with succinate were more susceptible to VSV infection than control mice. Taken together, these findings demonstrate that succinate suppresses the antiviral response of mice.

### Succinate Inhibits VSV-Triggered MAVS-IRF3 Signaling

**Figure 5:**
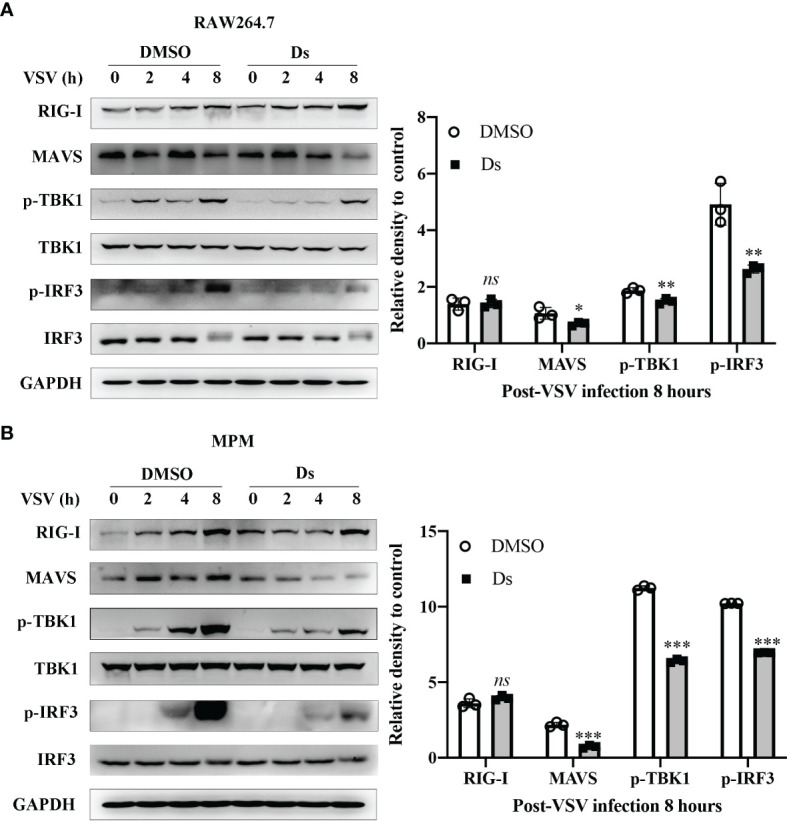
Succinate inhibits VSV-triggered MAVS-IRF3 signaling. **(A)** RAW264.7 cells were pretreated with 5 mM Ds or DMSO for 3 h followed infection by VSV (MOI = 1) for the indicated times. Cell lysates were immunoblotted with the indicated antibodies. The histogram exhibited the densitometric measurement of RIG-I/GAPDH, MAVS/GAPDH, p-IRF3/IRF3, and p-TBK1/TBK1 ratio relative to 0 h post VSV infection. **(B)** MPM were pretreated with 5 mM Ds or DMSO for 3 h followed infection by VSV (MOI = 1) for the indicated times. Cell lysates were immunoblotted with the indicated antibodies. Cell lysates were immunoblotted with the indicated antibodies. The histogram exhibited the densitometric measuring of RIG-I/GAPDH, MAVS/GAPDH, p-IRF3/IRF3, and p-TBK1/TBK1 ratio relative to 0 h post VSV infection. Data are shown as mean ± SD. Significance calculated using the unpaired *t*-test. *p < 0.05, **p < 0.01, ***p < 0.001. Representative images are from three independent experiments.

To explore the mechanisms on which succinate regulated the antiviral response, we examined the MAVS-IRF3 signaling which triggers antiviral response by sequentially activating the downstream axis for type I IFN production. The RAW264.7 cells and MPMs were pretreated with 5 mM Ds or DMSO and then infected with VSV (MOI = 1). The cells were lysed at 0, 2, 4, and 8 h post VSV infection, and the cell lysates were analyzed by Western blotting. As shown in **Figure 5A**, the MAVS, TBK1, and IRF3 phosphorylation was decreased in the Ds-pretreatment cells. Especially, at 8 h post-VSV infection, the MAVS expression in the Ds-pretreatment RAW264.7 cells was nearly 0.4-fold less than that in control cells. In the MPM cells, the MAVS, TBK1, and IRF3 phosphorylation was also decreased in the Ds-pretreatment cells (**Figure 5B**). Moreover, the MAVS expression in the Ds-pretreatment MPM cells was 0.8-fold less than that in DMSO-pretreatment cells. The PBS-treated cells were used as normal control. The expression levels of MAVS, TBK1, and IRF3 phosphorylation did not change in the PBS control group between the DMSO and Ds-pretreatment cells (**Figure S1**). Together, the succinate inhibits VSV-triggered activation of MAVS-IRF3 signaling.

### Succinate Suppresses the Activation and Aggregation of MAVS

**Figure 6:**
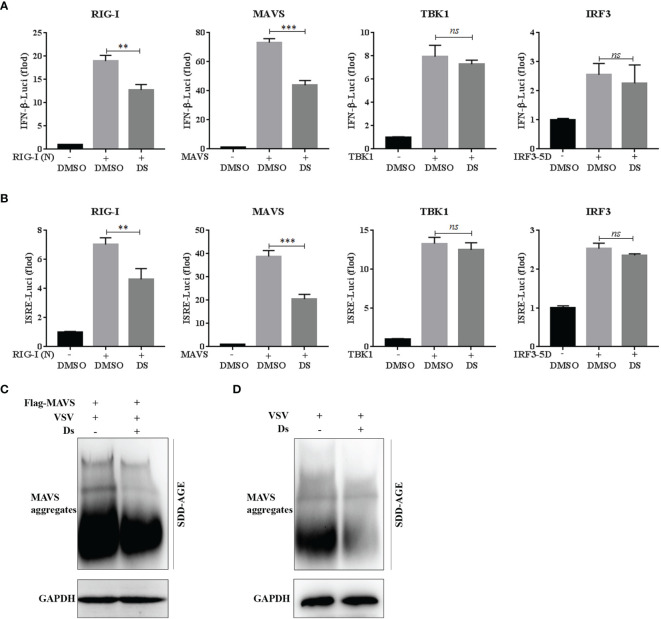
Succinate suppresses the activation and aggregation of MAVS. **(A, B)** 293T cells were pretreated with 5 mM Ds or DMSO for 3 h and then co-transfected with the IFN-β or ISRE luciferase reporter plasmid (90 ng), phRL-TK plasmid (10 ng), together with RIG-I (N), MAVS, IRF3-5D, or TBK1 plasmids (50 ng each). At 24 h after transfection, luciferase reporter assay was used to analysis of IFN-β **(A)** and ISRE **(B)** promoter activity. Data are shown as mean ± SD. Significance calculated using the unpaired *t*-test. **p < 0.01, ***p < 0.001, *ns*, no significant. **(C)** The HEK293T cells transfected with Flag-tagged MAVS plasmid were pretreated with Ds (5 mM) or DMSO for 3 h and then infected with VSV for 8 h. The cell lysates were prepared and analyzed by SDD-AGE using a MAVS antibody or SDS-PAGE using a GAPDH antibody. **(D)** The MPM cells were pretreated with Ds (5 mM) or DMSO for 3 h and then infected with VSV for 8 h. The cell lysates were prepared and analyzed by SDD-AGE using a MAVS antibody or SDS-PAGE using a GAPDH antibody.

To further identify the molecular target of succinate in the VSV-triggered innate immune response, dual-luciferase reporter assay was performed using IFN-β or ISRE luciferase reporter plasmid, phRL-TK plasmid, and RIG-I (N), MAVS, IRF3-5D, or TBK1 plasmids. At 24 h after transfection, IFN-β (a) and ISRE (b) promoter activities were analyzed using the luciferase reporter to detect the target of succinate in the RIG-I-MAVS signaling pathway. The data revealed that only the RIG-I-N- and MAVS-triggered IFN-β and ISRE luciferase activation could be inhibited by the pretreatment of succinate (**Figures 6A, B**). These results suggested that MAVS might be the target molecule of succinate in the RIG-I-MAVS signaling pathway. It was reported that viral infection could induce the formation of very large MAVS aggregates, which potently activate IRF3 in the cytosol (11, 18); thus, we investigated whether succinate could reduce the aggregation levels of MAVS induced by VSV infection. The Flag-MAVS plasmids were transfected into HEK293T cells. At 3 h post Ds or DMSO pretreatment, the cells were infected with VSV for 8 h, and the cell lysates were loaded onto SDD-AGE to analyze the aggregation of MAVS. The results showed that the aggregation of MAVS was decreased in the Ds-pretreated cells (**Figure 6C**). Similarly, in the Ds-pretreated MPM cells, VSV infection induces lower aggregation of MAVS than that in the DMSO-pretreated cells (**Figure 6D**). Collectively, succinate partially inhibits the activation and aggregation of MAVS.

## Discussion

In the present study, upon VSV infection, the genes of glucose transporter (GLUT) 1 and GLUT4 were increased, and isocitrate dehydrogenase 1 (IDH1) mRNA and α-ketoglutarate dehydrogenase (OGDH) mRNA production was decreased in mouse macrophages, indicating that VSV challenge leads to the glucose metabolism reprogramming. This finding is consistent with the recent studies showing that the changes in glucose metabolic pathways during RLR-induced type I IFN production are critical for host antiviral immune responses (15, 19, 20). Especially, our results showed that the tricarboxylic acid (TCA) cycle metabolite succinate was gradually decreased with the prolongation of VSV infection time in RAW264.7 cells. The phenomenon of decreased succinate was also shown in HEK293T cells transfected with poly(I:C), following the activation of RLR signaling (15). In contrast, succinate was shown to accumulate in the lipopolysaccharide (LPS)-stimulated macrophages during inflammation and in the *Salmonella typhimurium*-infected macrophages during intracellular infection (7, 21). Therefore, succinate plays diverse and important roles as an immunometabolite in innate immune response including anti-inflammatory response and antiviral response.

Our discovery that cell-permeable diethyl succinate inhibited the VSV-induced IFN-β production *in vitro* and *in vivo* particularly accelerated the death of the VSV-infected mice, indicating a negative regulatory function of succinate in innate antiviral response. This study reveals for the first time the role of succinate in cell and host immune response to viral infection, even though substantial evidence accumulated in recent years has highlighted the role of succinate in regulating inflammatory response induced by multiple stimuli. Interestingly, it remains controversial whether succinate can be considered as a harmful signal or a protective molecule in the inflammatory response. In macrophages exposed to LPS, in a mouse model of antigen-induced arthritis, and in a mouse model of TNBS colitis, succinate showed a role of harmful signal to increase the production of pro-inflammatory cytokines (4, 6, 7). However, there were some reports that showed that succinate acted as a protective molecule under different inflammatory conditions. Peruzzotti-Jametti et al. demonstrated that succinate activated calcium signaling and mitogen-activated protein kinase phosphorylation in rodent and human induced neural stem cells (iNSCs) and NSCs *via* mediating the activation of its receptor SUCNR1, and then elicited the anti-inflammatory function of iNSCs and NSCs by inducing the production of prostaglandin (PG) E2 (22). Moreover, the succinate-SUCNR1 signaling acted as a major driver of microbiota-triggered type 2 immunity in the intestine and was described as an anti-inflammatory mediator to regulate the metabolic response to obesity in macrophages (5, 23). So far, most studies on succinate in inflammatory response and other physiologic and pathological circumstances including in the muscle in response to exercise training and in the tumor microenvironment have focused on its receptor SUCNR1 expressed on various cell surfaces (24, 25). Thus, during inflammation, whether succinate acts as a pro-inflammatory driver or an anti-inflammatory signal might be due to the status of immune cells expressing SUCNR1.

Without considering the function of SUCNR1 in antiviral response, we demonstrated that succinate could inhibit the aggregation of mitochondrial antiviral-signaling protein (MAVS) and inhibit its expression and activation, thereby negatively regulating RIG-I-MAVS signaling and leading to decreased IFN-β production in VSV-infected mouse macrophages. In non-activated cells, MAVS is mainly as a monomer on the outer mitochondrial membrane in its monomeric form (11, 26, 27). Upon engagement of viral RNA, RIG-I and MAVS proteins undergo conformational changes leading to the activation of antiviral signaling (28). It was noteworthy that MAVS forms well-ordered, functional prion-like aggregates, which potently activate IRF3 in the cytosol (11). In this study, we found that succinate could suppress the formation of MAVS aggregates and then decrease the phosphorylation levels of IRF3. In recent years, increasing evidence showed that many molecules regulated antiviral immune response by mediating the formation of MAVS aggregates. For example, the mitochondrial ubiquitin ligase MARCH5 was reported to resolves MAVS aggregates during antiviral signaling (29). Besides, RNF34 promoted the autophagic degradation of MAVS aggregates to negatively regulate the innate immune response (30). Moreover, SARS-CoV-2 membrane glycoprotein M antagonizes the antiviral signaling by impairing MAVS aggregation and its recruitment of downstream TBK1 and IRF3 (31). Notably, glycolysis-derived lactate serves as a key metabolite and was shown to negatively regulate the RLR signaling by directly binding to the MAVS transmembrane domain and preventing MAVS aggregation (15). It is therefore reasonable to assume that the MAVS protein was the target molecule of succinate in the antiviral signaling.

In summary, we verified that succinate could inhibit cellular and host innate antiviral response by targeting MAVS and inhibiting the formation of MAVS aggregates. Our study provides not only a cross-link between the crucial immunometabolite succinate and antiviral immune response but also offers a crucial paradigm and strategy for the management of viral infection.

## Data Availability Statement

The raw data supporting the conclusions of this article will be made available by the authors, without undue reservation.

## Ethics Statement

The animal study was reviewed and approved by the National Institute of Health Guide for the Care and Use of Laboratory Animals with the approval of Shenzhen University (Approval Number: AEWC-20200316).

## Author Contributions

WC and YX conceived and designed the experiment. YX, XC, ZhuW, JQ, and HT performed the experiments. XZ, HW, QD, and ZhuW analyzed the data. WC and YX processed and typeset the figures. WC, YX, and ZheW wrote the manuscript. All authors contributed to the article and approved the submitted version.

## Funding

This work was supported by grants from the National Natural Science Foundation of China (Nos. U1801283, 31870908), Shenzhen Science and Technology Innovation Commission grant (No. JCYJ20180507182253653, JCYJ20190808172201639), and SZU Top Ranking Project (No. 86000000210) to WC.

## Conflict of Interest

The authors declare that the research was conducted in the absence of any commercial or financial relationships that could be construed as a potential conflict of interest.

## Publisher’s Note

All claims expressed in this article are solely those of the authors and do not necessarily represent those of their affiliated organizations, or those of the publisher, the editors and the reviewers. Any product that may be evaluated in this article, or claim that may be made by its manufacturer, is not guaranteed or endorsed by the publisher.

## Abbreviations

GPR91, G protein-coupled receptor; LPS, lipopolysaccharide; IFNs, type I interferons; PRRs, pattern recognition receptors; RIG-I, retinoic-acid inducible gene I; RLRs, retinoic-acid inducible gene I (RIG-I)-like receptors; MDA5, melanoma differentiation-associated gene 5; IRF3, interferon regulatory factor 3; HBP, hexosamine biosynthesis pathway; *O*-GlcNAc, *O*-linked β-N-acetylglucosamine; SAMHD1, histidine/aspartic acid domain-containing protein 1; MPM, mouse peritoneal macrophages; VSV, vesicular stomatitis virus; GLUT, glucose transporter; TCA, tricarboxylic acid; IDH1, isocitrate dehydrogenase 1; OGDH, α-ketoglutarate dehydrogenase; OXPHOS, oxidative phosphorylation; Ds, diethyl succinate; MOI, multiplicity of infection; CXCL-10, chemokine (C–X–C motif) ligand 10; ISG15, IFN-stimulated genes 15; PG, prostaglandin; iNSCs, induced neural stem cells.
