# Supplementary material for: Succinate Is a Natural Suppressor of Antiviral Immune Response by Targeting MAVS
Source: Front Immunol. 2022 Mar 2;13:816378. doi: 10.3389/fimmu.2022.816378 (PMC8924363; doi:10.3389/fimmu.2022.816378)
Supplement: Supplementary file 1 [file DataSheet_1.docx]

**Supplementary Information:**

**Succinate is a natural suppressor of antiviral immune response by targeting MAVS**

**Yue Xiao****^1^, Xinyi Chen^1^, Zhun Wang ^2^, Jiazheng Quan^1^, Xibao Zhao^1^, Haimei Tang^1^, Han Wu^1^, Qianqian Di^1^, Zherui Wu^1^, Weilin Chen^1,^***

^1^Department of Immunology, School of Medicine, Shenzhen University, Shenzhen, 518055, China.

^2^Technological Center, Changchun Customs 1301 Puyang Street, Changchun, Jilin, 130062, China,


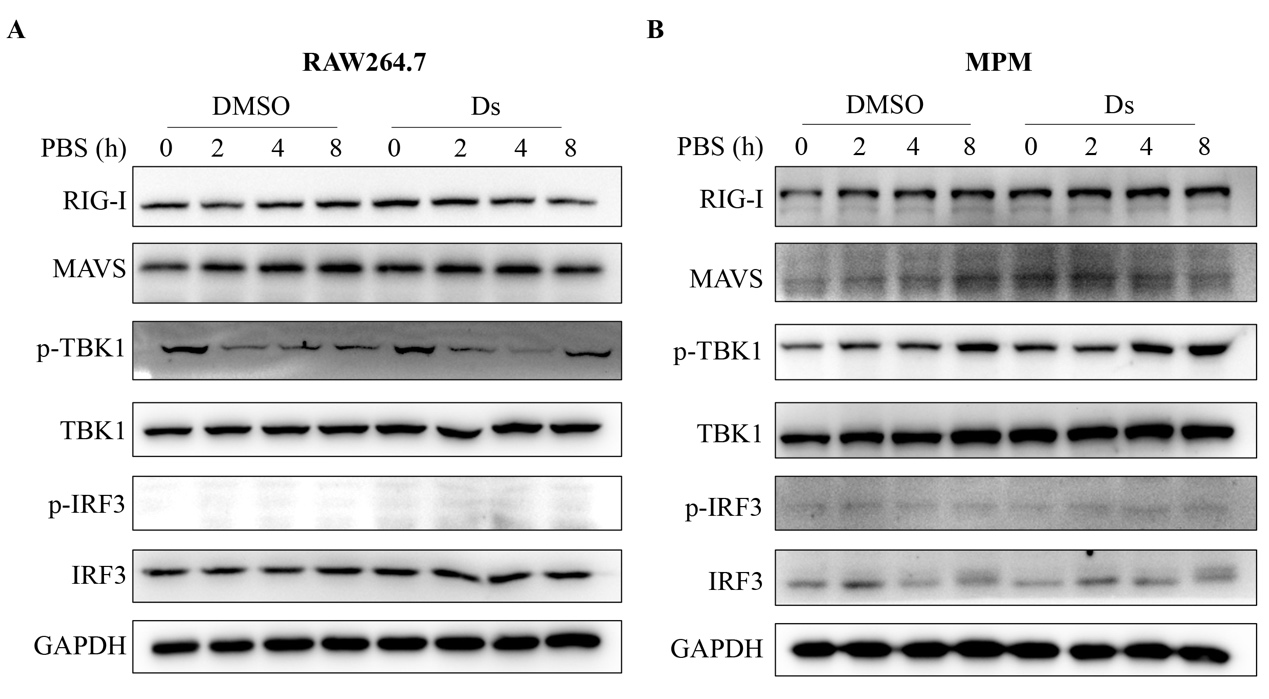


**Figure S1. The expression levels of MAVS-IRF3 signaling proteins in the mice macrophages treated with succinatee**. (**A**) RAW264.7 cells and (**B**) MPM were pretreated with 5 mM Ds or DMSO for 3 h followed treatment with PBS the indicated times. Cell lysates were immunoblotted with the indicated antibodies.
